# Tai Chi for type 2 diabetes mellitus: A protocol for systematic review

**DOI:** 10.1097/MD.0000000000018853

**Published:** 2020-01-24

**Authors:** Xixi Chen, Furong Zhang, Jian Li, Xiujuan Zhou, Mingsheng Sun, Xicen Liu, Danyang Liu, Xiaoyu Shen, Rongjiang Jin

**Affiliations:** a College of Health Preservation and Rehabilitation, Chengdu University of Traditional Chinese Medicine, Chengdu city, Sichuan province; b School of Kinesiology, Shanghai University of Sport, Shanghai city, China; c College of Clinical Medicine, Chengdu University of Traditional Chinese Medicine, Chengdu city, Sichuan province; d College of Acupuncture and Tuina, Chengdu University of Traditional Chinese Medicine; e Nuclear Industry 416 Hospital, Chengdu city, Sichuan province, China.

**Keywords:** meta-analysis, Tai Chi, Type 2 diabetes, umbrella review

## Abstract

**Background:**

Tai Chi is gaining an increasing popularity in rehabilitation management of chronic conditions. Yet no consensus has reached on its efficacy and safety of type 2 diabetes despite that several systematic reviews (SRs) were published on this topic. Therefore, we will conduct an overview to critically evaluate current SRs and implement an updated metaanalysis with recently published randomized controlled trials (RCTs).

**Methods:**

A systematic literature search of relevant RCTs-based SRs will be conducted in electronic databases including Medline, Embase, the Cochrane Library, the Chinese National Knowledge Infrastructure, the Chinese Biomedical Literature Database from their inceptions to search date without language restrictions. Eligible SRs will be methodologically assessed by the assessment of multiple SRs 2 and Risk of Bias in SRs tool and their RCTs included will be extracted for further evidence synthesis. To update current meta-analysis on this topic, a supplementary search will be implemented for related newly emerged RCTs. Cochrane risk of bias assessment tool will be applied for RCTs quality evaluation. The grading of recommendations assessment, development and evaluation will be utilized for evidence quality assessment of outcomes. Study characteristic information on participants, interventions, outcomes, comparisons and conclusions will be described in detail. Review Manager V5.3 will be used for risk of bias assessment and Stata 14.0 for meta-analysis and sensitivity analysis.

**Results:**

The study results will be disseminated through a peer-reviewed journal publication or conference presentation.

**Conclusions:**

This study finding will provide an updated evidence of Tai Chi for patients with type 2 diabetes mellitus (T2DM), thus to help inform clinical physicians, T2DM patients and their families to develop better rehabilitation plans and to draw more attention of decision-makers in exercise rehabilitation related policy-making.

This study protocol has been applied for registration on PROSPERO platform (https://www.crd.york.ac.uk/PROSPERO/), with an assigned ID: CRD42019140988

## 1 Introduction

Type 2 diabetes mellitus (T2DM), a common chronic noncommunicable condition in clinic, is featured by physical metabolic disorder and manifested as absolute hyperglycemia and relative insulin insufficiency (caused by inadequate insulin secretion or insulin receptor insensitivity). Without well management, diabetic people are more likely to develop with complications like coronary artery disease,^[1]^ hypertension, kidney failure,^[2]^ nephropathy, ketoacidosis,^[3]^ hyperosmolar coma, severe lower-limb infections and so on. However, many diabetic patients are even unaware of such complications.^[4]^

Though not contagious, it has already become a pandemic, drawing increasing attention and effort to solve this health crisis worldwide. According to the 8th IDF Diabetes Atlas, diabetes has affected 425 million people in 2007, and this number will rise to 629 million by 2045 as estimated. Among all diabetic cases, T2DM accounts for 90% approximately.^[5,6]^ China, as the most populated nation, its estimated diabetes prevalence was 10.9% according to a 170,287-participant-invloved cross-sectional survey nationwide in 2013,^[7]^ with an estimate of 113.9 million and 493.4 million Chinese adults with diabetes and prediabetes respectively based on sample-weighting project.^[8]^ The health expenditure on diabetes reached USD 850 billion in 2017 covered an expanded age range from 18 to 99 years old based on the 8th Diabetes Atlas. The originally high incidence and prevalence is still on the increase, posing great health threats and heavy socioeconomic burdens globally.

Currently, recommended managements for T2DM include losing weight, adopting a healthy lifestyle (eat healthily and exercise properly), antidiabetic medication, and insulin; besides, bariatric surgery is optional if necessary. Among these interventions, exercise therapy has been gaining an increasing popularity and been recommended in T2DM management, for regular physical activity can improve blood glucose control as well as lipids indexes, blood pressure, cardiovascular diseases and life quality.^[9]^ Tai Chi, originated from Chinese martial art, has now developed into a mind-body exercise for rehabilitation and health preservation. It integrates gentle and slow physical movement and meditation methodically, thus to harmonize *Yin* and *Yang* in the body. Different types are classified based on movement number, posture and feature of each style, and named after its creator, mainly including *Yang* style, *Wu* style, *Chen* style, *Sun* style and *Wu* style. Tai Chi practicing requires no additional equipment but bare hands, thus making it a convenient and economical rehabilitation intervention. There is a trend that Tai Chi has been applied more and more widely in clinical practice as an exercise therapy. Benefits of *Tai Chi* on chronic conditions including Parkinson disease,^[10]^ fibromyalgia,^[11]^ and knee osteoarthritis^[12]^ have been reported in well-known public medical journals like BMJ, NEJM, and so on. It has also been used in T2DM management. Several systematic reviews (SRs) on efficacy and safety of Tai Chi on T2DM were done, yet no consensus reached.^[13–15]^ As a promising intervention, its effectiveness needs to be confirmed. Therefore, this study will be implemented to critically evaluated the quality of current published SRs and make an updated meta-analysis due to newly emerged studies.

## 2 Methods and analysis

This protocol will be reported under the guidance of the preferred reporting item for SR and metaanalysis protocols guidelines.

**Protocol registration** This study protocol has been applied for registration on PROSPERO platform in advance (https://www.crd.york.ac.uk/PROSPERO/), with an assigned ID: CRD42019140988.

### 2.1 Eligibility criteria

The classical PICOS (participant, intervention, comparison, and study design) approach has been implemented in this study.

(1) Participants: Patients diagnosed with type 2 diabetes mellitus bases on clinical criteria. Studies on patients with type 1 diabetes, latent autoimmune diabetes in adults or glucose disorder cause by other conditions like pregnancy, hyperthyroidism, acromegaly, pheochromocytoma, Cushing syndrome, stress stimulations will be excluded.
(2) Interventions: Bare-handed Tai Chi Quan exercise of different styles will be taken as interesting interventions. Tai Chi sword, Tai Chi ball and other forms of exercise named after Tai Chi will be excluded. Combined interventions will be allowed if all groups received the same basic intervention.
(3) Comparisons: Include placebo; no treatment; pharmacological therapies; nonpharmacological interventions besides Tai Chi. Trials that compare different styles of Tai Chi will be excluded.
(4) Outcomes: The primary outcomes will be glycosylated hemoglobin and fasting blood glucose, for these 2 indexes can effectively and sensitively reflect the situation of glucose control among patients with T2DM. Secondary outcomes will include health related quality measurements like SF-36, body mass index, lipids-related indexes, and adverse events, and these measurements are closely related with patients metabolism situations, safety and life quality.
(5) Study design: Only randomized controlled trials (RCT)-based SRs will be included in the overview, and RCTs with both parallel and crossover designs will be included in the updated meta-analysis. Non-RCTs, cluster randomized trials, array studies, guidelines, editorials, and comments will be excluded.

### 2.2 Information sources and search strategy

We will carry out a systematic literature search of relevant RCT-based SRs in electronic databases including medline via Pubmed, Embase, web of science, the Cochrane Library, the Chinese National Knowledge Infrastructure, the Chinese BioMedical Literature Database and Wanfang database from their inceptions to commencement date without language restrictions. If with duplicated versions, the latest one will be preferred. To guarantee the newly emerged RCTs will be included in the updated meta-analysis, a supplementary search will be conducted from the search date in the latest SR to commencement date of this study. The search strategies will be developed with the guidance of an experienced SR author, with an integration of medical subject headings terms and keywords. A preliminary search strategy is provided in Tables 1 and 2, which will be adapted based on different syntax-related requirements of databases. Besides, the ongoing studies (including SR and RCT) will be searched in registration platforms like PROSPERO database, World Health Organization International Clinical Trials Registry Platform and clinicaltrials.government. Their authors will be contacted by email to identify additional data if necessary. The references of relevant SRs will be screened also, and a search of professional endocrinological journals and meeting abstracts will be manually checked for potentially relevant studies.

**Table 1:**
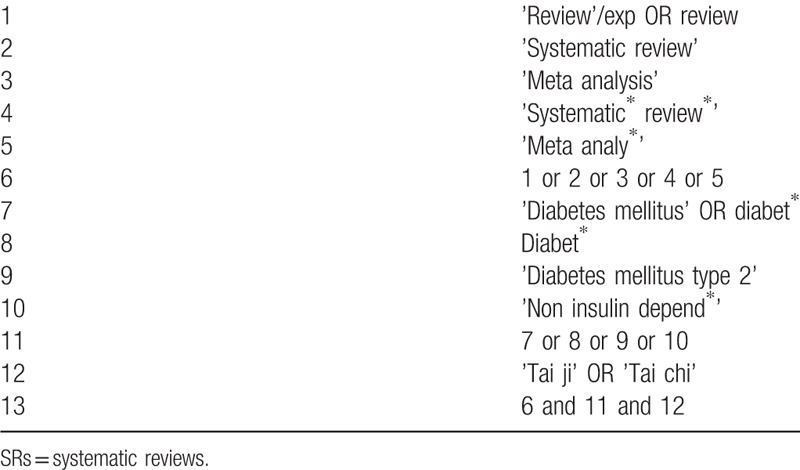
Preliminary search strategy of SRs in Embase.

**Table 2:**
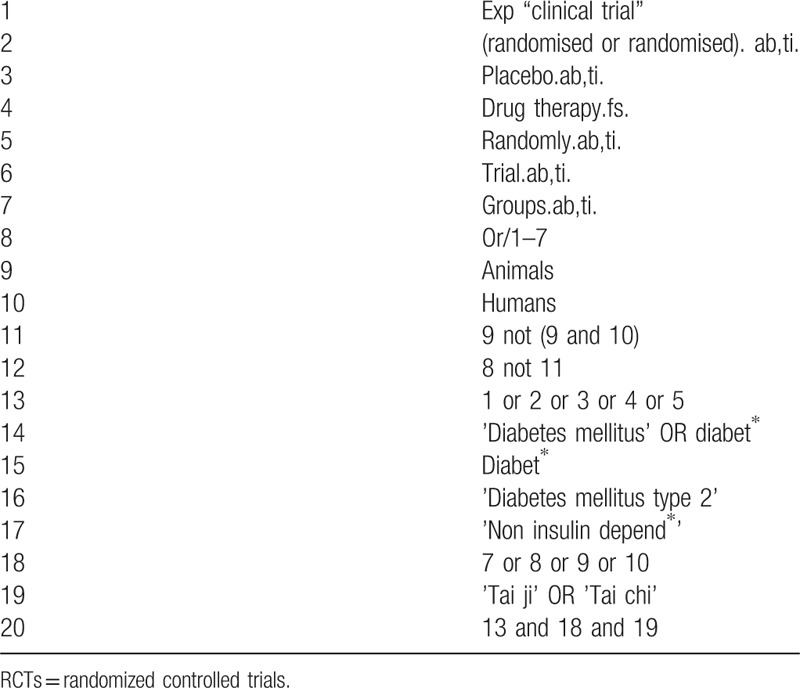
Preliminary search strategy of RCTs in Embase.

### 2.3 Study identification and data extraction

**Figure 1:**
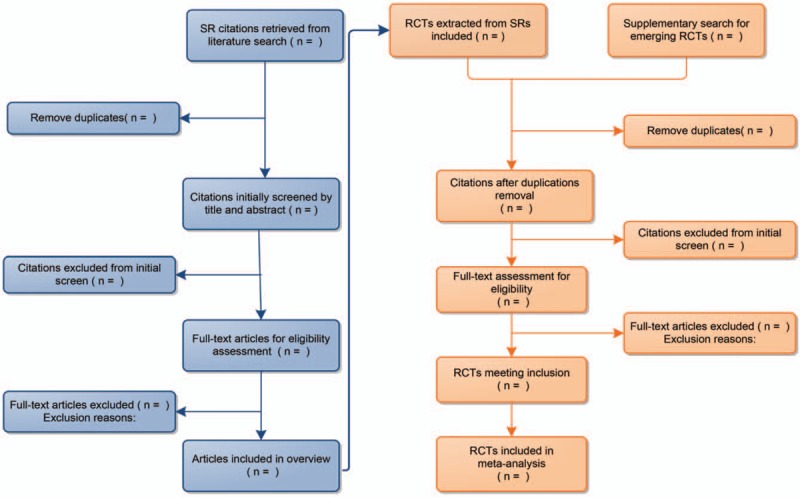
Flow diagram of the study design. This flow diagram is based on PRISMA framework, which shows the whole process of literature (RCT and SR) and meta-analysis for this research topic, including records retrieving, screening, inclusion, exclusion with reasons and articles included in final qualitative and quantitative analysis. RCTs = randomized controlled trials, SR = systematic review.

All retrieved citations will be imported and managed in Endnote X7. Two independent authors (FRZ and JL) will initially review all citations by title and abstract, discarding duplicates and downloading full texts of potentially eligible citations for further evaluation. After full-text screen, studies meeting predefined inclusion criteria will be included. The excluded articles will be listed in a form with detailed exclusion reasons. The whole process of study identification will be displayed in a PRISMA-based flow chart (see Fig. 1)

A standard pre-designed form will be utilized for date extraction, including items of general information (first author, country, year of publication), clinical features (number of centers and the participants, details of interventions, comparisons, outcomes and brief conclusions) and methodological characteristics (study design, sample size, randomization, and allocation, blinding). RCT authors will be contacted for missing data acquisition. Discrepancies between the 2 reviewers will be settled by team discussion or the introduction of a third reviewer (RJJ).

### 2.4 Risk of bias assessment

All eligible SRs will be methodologically evaluated by the assessment of multiple systematic reviews 2 (AMSTAR 2) and risk of bias in systematic reviews (ROBIS) tools. AMSTAR 2 is an updated tool for SR quality assessment with 7 critical domains, which has been introduced and applied in our previous publication^[16]^ ROBIS (access at http://www.robis-tool.info) is a tool for risk of bias assessment of SRs, which contains 3 phases: assess relevance (optional), identify concerns within review-making process and judge risk of bias in the review.^[17]^ RCTs included in the updated review and meta-analysis will be assessed with the Cochrane Collaboration's risk of bias tool (https://training.cochrane.org/handbook), which focuses on 6 domains including sequence generation, allocation concealment, blinding, incomplete data, selective reporting and other bias. Two independent authors (FRZ and XXC) will accomplish this work. Any disagreement during this procedure will be settled by discussion among authors or through the introduction of a third experienced reviewer. Risk of bias assessment of RCTs will be done with Cochrane Review Manager 5.3; For SRs, the assessment will be done with word-filed checklists of AMSTAR 2 and ROBIS.

### 2.5 Statistical analysis

Data for analysis will be extracted and managed in an excel form. To calculate the summary effect size, Continuous data will be expressed as standardized mean difference and categorial data as relative ratio with 95% confidence intervals. SRs included in the umbrella review will be synthesized qualitatively, reporting mainly on methodology quality, interventions, and outcomes; While RCTs included in the updated SR will be pooled with metaanalysis method if with enough homogeneity. Either random or fixed effect model will be selected according to the degree of heterogeneity. The heterogeneity of included RCTs will be appraised with *Q* test, and quantitively displayed by *I*^2^ statistic value, and the computational formula is *I*^2^ = (*Q*-*v*)/*Q*, in which *v* represents degree of freedom, resulting from study number minus 1. If *I*^2^ ≤ 50%, heterogeneity is considered acceptable, and a fixed-effect model will be selected; If *I*^2^ > 50%, it implicates the existence of substantial heterogeneity, subgroup and sensitivity analysis will be carried out to investigate potential sources of heterogeneity. For instance, if enough studies take different style of Tai Chi as interventions, they will be stratified by disparate style for subgroup analysis. After a series of processing mentioned above, if *I*^2^ value is still greater than 50%, a randomized-effect model will be applied; But if it is too significant like *I*^2^ > 70%, a narrative synthesis will be conducted instead of a metaanalysis. Besides, publication bias will also be demonstrated with a funnel plot if with more than 10 studies included. Stata 14.0 will be used for meta-analysis, sensitivity analysis, and plots drawing.

### 2.6 Grading of recommendations assessment development and evaluation (GRADE) -based evidence quality assessment

To promote understanding and application of evidence, the GRADE framework will be employed to evaluate the evidence quality and recommendation strength regarding main outcomes of meta-analysis. Furthermore, confidence in each outcome estimate will be downgraded from high to moderate, low or very low, depending on GRADE quality assessment results from 5 domains involving study limitations, inconsistency, indirectness, imprecision, and publication bias. This procedure will be implemented with GRADE pro 3.6 software by two authors separately. Disagreements will also be settled with method mentioned previously.

## 3 Discussion

Systematic review, though topped the evidence pyramid, the quality of itself may vary due to methodological deficits and other factors. Thus, it is important to assess already published ones before conducting a new SR especially with controversies in a certain field. The results of this study will provide a systematically methodological evaluation of currently published SRs on efficacy and safety of Tai Chi for T2DM, and a new synthesis of evidence on this topic with an updated metaanalysis. Back to the point, we expect this review result would interest and benefit clinical physicians, rehabilitation specialist, T2DM patients and their family, as well as policymakers in medical decision-making.

## Acknowledgment

All authors would like to thank the library of Chengdu University of TCM for providing literature resources.

## Author contributions

**Analysis planning:** Furong Zhang, Jian Li

**Conceptualization:** Furong Zhang, Xixi Chen, Rongjiang Jin,

**Data curation:** Xixi Chen, Xiujuan Zhou, Xicen Liu.

**Draft manuscript:** Xixi Chen, Furong Zhang, Jian Li

**Investigation:** Mingsheng Sun, Xiujuan Zhou, Danyang Liu.

**Manuscript editing:** Xiaoyu Shen, Rongjiang Jin

**Methodology:** Jian Li, Xixi Chen, Furong Zhang.
